# Electronic Properties of TiO_2_ Nanoparticles Films and the Effect on Apatite-Forming Ability

**DOI:** 10.1155/2013/139615

**Published:** 2013-05-12

**Authors:** Johanna Löberg, Jenny Perez Holmberg, Ingela Mattisson, Anna Arvidsson, Elisabet Ahlberg

**Affiliations:** ^1^DENTSPLY Implants, Box 14, 431 21 Mölndal, Sweden; ^2^Department of Chemistry and Molecular Biology, University of Gothenburg, 412 96 Gothenburg, Sweden

## Abstract

Nanoparticle-covered electrodes have altered properties as compared to conventional electrodes with same chemical composition. The changes originate from the large surface area and enhanced conduction. To test the mineralization capacity of such materials, TiO_2_ nanoparticles were deposited on titanium and gold substrates. The electrochemical properties were investigated using cyclic voltammetry and impedance spectroscopy while the mineralization was tested by immersion in simulated body fluid. Two types of nucleation and growth behaviours were observed. For smooth nanoparticle surfaces, the initial nucleation is fast with the formation of few small nuclei of hydroxyapatite. With time, an amorphous 2D film develops with a Ca/P ratio close to 1.5. For the rougher surfaces, the nucleation is delayed but once it starts, thick layers are formed. Also the electronic properties of the oxides were shown to be important. Both density of states (DOS) in the bandgap of TiO_2_ and the active area were determined. The maximum in DOS was found to correlate with the donor density (*N*_*d*_) and the active surface area. The results clearly show that a rough surface with high conductivity is beneficial for formation of thick apatite layers, while the nanoparticle covered electrodes show early nucleation but limited apatite formation.

## 1. Introduction

Nanocrystalline films composed of sintered nanoparticles of TiO_2_ have interesting properties and have been studied for potential use in photoelectrochemical solar cells, electrochromic windows, and batteries with Li-intercalation [1–7]. The advantages compared with conventional materials are the much larger internal surface accessible due to electrolyte penetration of the porous framework, efficient charge separation, and ion conduction since the transport distance for charge carriers to travel becomes short in the nanocrystallites [8, 9]. A disadvantage noted for these applications is the large density of states. These states take part in electron-hole recombination processes and electron transfer reaction at the oxide-electrolyte interface, if located in the bandgap. However, the latter property can be regarded as an advantage for adsorption of redox-active compounds and is therefore of special interest for dental implant applications, which the present study is focused on. It has, for example, been shown that serum proteins such as fibrinogen adsorb on the titanium surface through a chemisorptions process, that is, involving electron transfer [10]. The ability of a material to attract biomolecules, specific proteins, and blood platelets and thus trigger the blood coagulation are key factors to induce bone formation directly on an implant surface [11, 12]. The electronic behaviour of TiO_2_ has also been shown to affect the thrombogenicity of the material [13–15], and one explanation could be the semiconducting behaviour of fibrinogen which is important in the blood coagulation pathway. Fibrinogen has a band structure that fits into the bandgap of TiO_2_ and by altering the gap size and/or the *E*_*F*_ placement of TiO_2_, the protein adsorption and following decomposition of the fibrinogen could be changed [13–15]. It is therefore important to study the electronic properties of different titanium oxide layers and in particular to estimate the density of states in the bandgap of TiO_2_.

The aim of the present study is to investigate the effect of surface roughness, morphology, and electronic properties on the apatite-forming ability of surfaces with different nanostructures. For this purpose, surfaces with well-characterized nanoparticles were formed using spin coating. Three different suspensions of nanoparticles were used with 8 and 22 nm clean TiO_2_ particles individually dispersed and commercial P25 particles with an individual size of ~30 nm. The P25 dispersion contained also larger aggregates that were transferred to the surface. A turned Ti surface was used as reference, and a nanostructured surface formed by etching in acid solution was used for comparison. Surface roughness, morphology, and composition were determined using AFM, SEM, EDX, and XRD, respectively. The electronic properties were determined using cyclic voltammetry and impedance spectroscopy and correlated with the bioactivity measured by immersion in simulated body fluid for different times.

## 2. Materials and Methods

### 2.1. Sample Preparation

Titanium dioxide (TiO_2_) nanoparticles were synthesized by controlled hydrolysis of TiCl_4_ to obtain clean surfaces. TiO_2_ particles of different sizes were obtained by synthesis at 0°C but with dialysis and storage of the colloidal dispersions done at 0°C for 8 nm and 20°C for 22 nm sized particles. The dialysis step is important to avoid agglomeration, and the resulting suspensions consist mainly of single nanoparticles. Details about the synthetic procedure and properties of the suspensions are given elsewhere [18, 19]. The two particle sizes were chosen based on previous work where fundamental properties have been addressed and shown to be size dependent [19, 20]. Commercial TiO_2_ (Degussa P25), with an individual particle size in the range of 30–80 nm and 4 : 1 ratio of anatase and rutile [21], was carefully washed to remove organic surface species and treated in ultrasonic bath to get a dispersed solution. However, dynamic light scattering showed the existence of larger aggregates, and it was not possible to fully disperse the system. The particles were spin coated onto Ti (grade 4 discs with a turned surface, diameter 1.1 cm) or Au disks (polished with SiC paper (4000), diameter 1.1 cm). Typically, 100 *μ*L of particle suspension (~12.5 g/L for the 8 and 22 nm particles and 10 g/L for P25) was deposited on the electrode surface. The rotation rate was initially 1500 rpm (30 s) and was increased to 3000 rpm (60 s) and then back to 1500 rpm before it was turned off. This procedure was repeated once. After spin coating, the samples were rinsed with deionized water and left to dry before use. As a complement to the nanoparticle-coated surfaces, the nanostructured TS + AT-I (turned surface treated with oxalic acid and hydrofluoric acid) surface was included in this study. Details for the AT-I treatment have been published elsewhere [16].  Table 1 shows a description of the samples used.

### 2.2. Surface Chemistry

A Siemens D5000 powder diffractometer which utilizes CuK*α* radiation (*λ* = 1.54056 Å) was used for identification of crystalline phases. X-ray diffraction was measured at different incidence angles in order to obtain information from different depths of the samples. For XPS analysis a Quantum 2000 ESCA Scanning Microscope (Physical Electronics, USA) with an X-ray source of monochromatic AlK*α* was used.

### 2.3. Surface Topography

High resolution SEM images were recorded using Leo Ultra 55 FEG SEM operated at 1 kV. Surface roughness analysis was performed by using atomic force microscopy (AFM) (Nanoscope Multimode IIIa, Digital Instruments). Tapping mode AFM measurements were performed at three points per sample and at three different scan sizes, 10 × 10, 5 × 5, and 3 × 3 *μ*m (scan frequency 0.8 Hz, 512 lines). The AFM data were imported into MeX [22] (Alicona Imaging GmbH) software where roughness analysis and calculation of 3D-surface roughness parameters were performed. By using multiple scan sizes and by applying a Gaussian filter of different sizes in the MeX software, information about topographical features ranging from 10 *μ*m to 150 nm are received, for details see [23].

### 2.4. Electrochemical Characterisation

Cyclic voltammetry and impedance measurements were performed by using a Gamry Reference 600 Potentiostat/Galvanostat/ZRA. The electrochemical measurements were done in a specially designed three-electrode cell used for stationary conditions. The sample is placed at the bottom of the cell with the turned surface towards the electrolyte. A large Pt counter electrode is concentrically placed around the sample to assure optimal current distribution, and the reference electrode is placed in the middle of the cell. This cell configuration is useful for all types of planar samples and shows good characteristics in impedance measurements. All potentials are referred to the Ag/AgCl (sat KCl, *E* = 0.197 V versus SHE) reference electrode.

#### 2.4.1. Cyclic Voltammetry (CV)

Cyclic voltammetry was performed in 0.1 M KOH in a broad potential range. The sweep rate was kept constant at 50 mVs^−1^.

#### 2.4.2. Electrochemical Impedance Spectroscopy (EIS)

Electrochemical impedance measurements were performed in deaerated 0.5 M H_2_SO_4_, and a continuous low purge with N_2_ gas was maintained during the impedance measurements. Impedance spectra were recorded at a constant potential in the frequency range 1 kHz to 10 mHz with 9 point/decade and an amplitude of 10 mV rms. The potential was stepped by 50 mV from +1 to −0.5 V with a waiting time of 300 s before the next spectra were recorded. The impedance data were fitted by using equivalent circuits consisting of one or two time constants in series with the solution resistance (*R*_sol_). Each time constant consists of a constant phase element (CPE) in parallel with a resistance related to the oxide. Different models have been used to calculate the effective capacitance depending on the system studied [24, 25]. It has been demonstrated that for lateral distribution of time constants, the solution resistance is included in the calculations, while for a distribution of time constant perpendicular to the surface, it can be omitted. For the system studied here, where a porous oxide is formed and electrolyte can penetrate most of the layer, distribution of time constants occurs laterally due to the porosity of the surface, and the effective capacitance has therefore been calculated using

(1) C=[Q·(1Rsol+1Rfilm)α−1]1/α,

where *R*_sol_ is the solution resistance, *R*_film_ is related to the oxide film resistance, and *α* is the dispersion factor for the CPE element. The effective capacitance was used to estimate the electric properties of the semiconducting titanium dioxide layer using the well-known Mott-Schottky relationship (2):

(2) 1Csc2=(2εrε0eNd)(E−Efb−kTe),

where *C*_sc_ is the space charge capacitance, *ε*_*r*_ the dielectric constant of TiO_2_, *ε*_0_ the dielectric constant of vacuum, *N*_*d*_ the number of charge carriers, e the charge of the electron, *E* the applied potential, and *E*_fb_ the flat band potential. Here it is assumed that the double-layer capacitance is much higher than the space charge capacitance. According to (2), a linear dependence is expected from which the number of charge carriers can be obtained from the slope and the flat band potential from the intercept.

### 2.5. Immersion in Simulated Body Fluid (SBF)

The SBF solution was prepared at 37°C according to the revised SBF recipe described in [26]. The pH of the solution was set to 7.00 ± 0.05 using 1 M NaOH, and the prepared SBF solution was used fresh. Samples were individually immersed and mounted with the treated surface upside down in 40 mL SBF solution. 9 Samples per category were immersed at 37.0°C, and 3 samples per category were evaluated after 12 h, 72 h, and 1 week of immersion. XRD analysis was performed at one sample per category and immersion time to investigate the chemical composition of the sample. SEM (ESEM XL30, FEI Company) and EDX (Apollo 14, EDAX) analyses were performed at 3 points per sample for all sample types to evaluate the amount and morphology of the formed apatite. SEM settings used were 10 kV, working distance 10 mm, and analysis area 126 × 102 *μ*m.

## 3. Results and Discussion

### 3.1. Surface Chemistry

**Figure 1:**
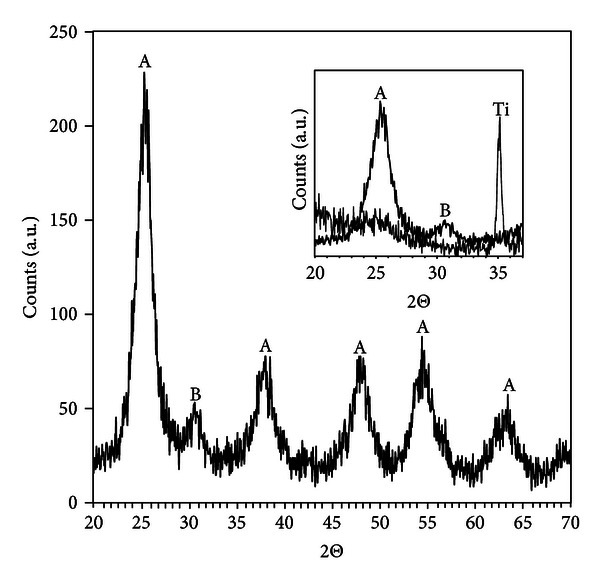
X-ray diffraction of TiO_2_-synthesised nanoparticles with 8 or 22 nm size. The particles were dried at 120°C for 16 h prior to the analysis. Diffraction peaks marked with A correspond to anatase while B stands for brookite. The inset shows enlargement of the diffraction pattern for the particles dispersed on Ti (thin line). The film was washed and allowed to dry at room temperature overnight.

Figure 1 shows X-ray diffraction of the nanoparticles in powder form obtained from suspensions containing 8 or 22 nm particles. The main phase is anatase but with a small contribution from brookite as shown by the reflection at 2*θ* = 30.8°. The broad diffraction peaks indicate that the particles consist of smaller crystallites (~4 nm), and growth studies have shown that the particles are formed by slow aggregation of initially formed precipitates of this size [18]. In the inset of Figure 1, the diffraction pattern for the particles attached to titanium is shown. Since the nanoparticle layer is thin, the signal is very weak but the main anatase peak can be observed. This shows that the phase of the particles remains after deposition and drying. The P25 particles attached to titanium show typical diffraction pattern with a ratio between anatase and rutile of ~4/1 [22]. For the TS + AT-I modification, no diffraction peaks from the oxide can be observed, which indicates that the precipitated layer is amorphous or too thin to be detected [16].

XPS on the nanoparticle films shows pure TiO_2_ with traces of chloride. No signal for Ti metal was observed, which indicates that the films fully cover the surface. Lower valent titanium ions were not observed in contrast to other studies showing Ti(III) at the surface of nanoparticles formed by other synthetic routes [27]. The carbon signal is similar for all samples and is related to surface contamination. XPS for the AT-I modification has been studied in detail elsewhere and shows that the oxide film may contain oxalate from the formation process [16]. In this case, a small Ti metal signal is observed illustrating that some parts of the surface have a thin oxide film.

### 3.2. Surface Topography

**Figure 2:**
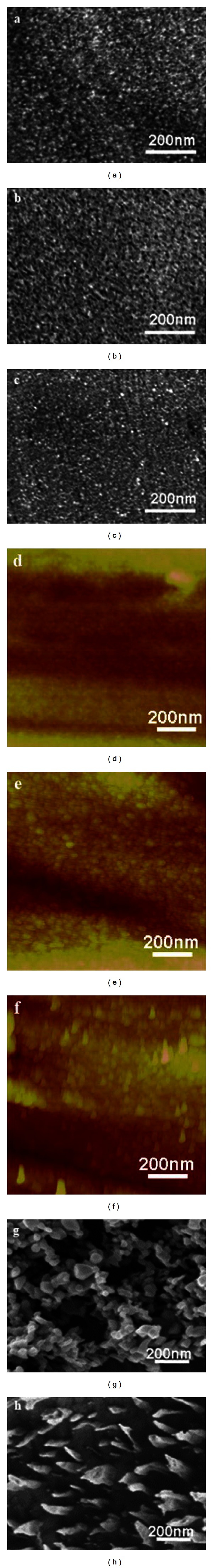
SEM images for the (a) TS + 8 nm, (b) TS + 22 nm, (c) Au + 22 nm, (g) TS + P25, and (h) TS + AT-I surfaces. AFM images for the (d) TS + 8 nm, (e) TS + 22 nm, and (f) Au + 22 nm surfaces.

Figure 2 shows high-resolution SEM images of surfaces coated with 8 nm (TS + 8 nm) and 22 nm (TS + 22 nm) and (Au + 22 nm) TiO_2_ particles. All three surfaces look fairly smooth and in order to further investigate the surface roughness also AFM images were recorded, middle row of Figure 2. In AFM, the TS + 8 nm surface looks smoother than the TS + 22 nm and Au + 22 nm surfaces, which is also quantified by surface roughness measurements, see Figure 2. From the SEM and AFM images of these surfaces it seems like the underlying substrate is fully covered. This is not the case for the two other modifications used in the present work. The suspension of P25 contained agglomerates of particles, and these were transferred to the Ti surface during the spin coating procedure. As a result, the surface roughness is high but in between the turned surface is visible, Figure 2(g). For the TS + AT-I surface etched in oxalic acid and hydrofluoric acid sequentially, rather large precipitates form with a very thin oxide layer in between, Figure 2(h). In contrast to the spherical nanoparticles, the precipitates on the TS + AT-I surface can be regarded as rods with a height of 0.45 *μ*m (determined from the average of maximum height of 5 peaks and valleys, *S*_10*z*_).

Topographical analysis of the coated and nanostructured surfaces was performed by AFM analysis using overlapping scan sizes and a Gaussian filter of different sizes to obtain information of surface features in the range of 10 to 0.150 *μ*m as described earlier [23]. The 3D surface roughness parameters were calculated using the MeX software [22], and values for three different parameters are listed in Table 2.

The *S*_*a*_ value (average height) is significantly larger for the surfaces TS + P25 and TS + AT-I than for the nanoparticles-coated surfaces and the turned surface. Both surface treatments induced additional surface structures on top of the turned surface without fully covering it, Figure 2. The coatings containing 8 and 22 nm particles completely covered the turned surface with particles, which in turn caused a decrease in surface roughness since the turning tracks are covered, Figure 2 and Table 2. The same trend is observed for the gold substrate, where the *S*_*a*_ value decreases after coating with 22 nm particles. There is only a slight difference between the *S*_*a*_ values for the TS + 8 nm and TS + 22 nm surfaces, but the lower value obtained for the smaller particles indicates that the curvature of the particles are reflected in the surface roughness. The root mean square of the slope (*S*_*dq*_) has been shown to correlate with the interface shear strength and is thus an important parameter to investigate for dental implant applications [28, 29]. From a biomechanical point of view, a large *S*_*dq*_ value is desired giving the TS + P25 surface an advantage. The trend in *S*_*dr*_ (developed interfacial area) values follows the same trend as the *S*_*a*_ and *S*_*dq*_ values with the smallest values obtained for the surfaces coated with nanoparticles. Analysis of roughness parameters calculated from 5 × 5 *μ*m and 3 × 3 *μ*m scan sizes shows lower absolute numbers but the trends for the different surfaces are the same as shown in Table 2.

### 3.3. Electrochemical Characterisation

#### 3.3.1. Cyclic Voltammetry

**Figure 3:**
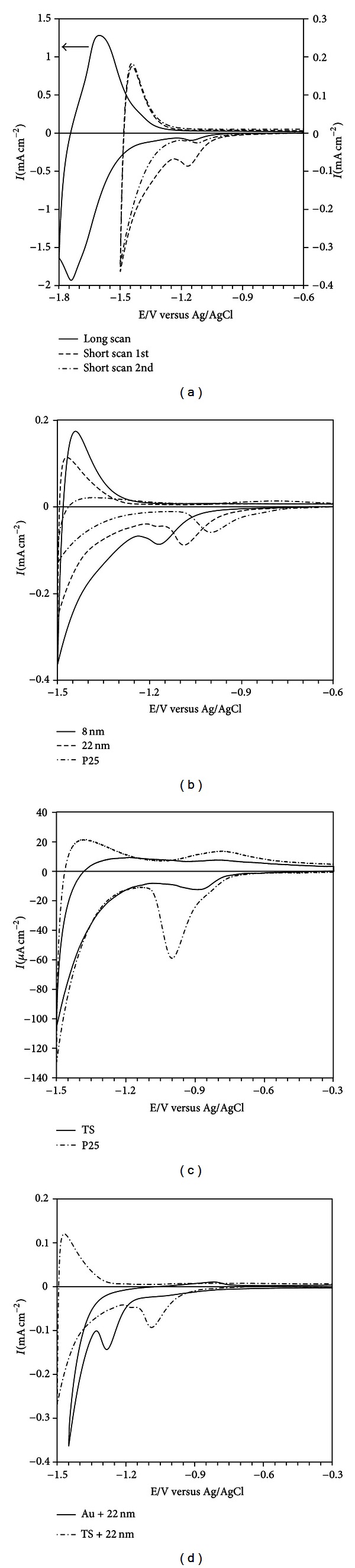
Cyclic voltammetry on different surfaces in deaerated 0.1 M KOH. (a) Surface coated with 22 nm TiO_2_ particles. The short and long scans are made on different samples. Note that the scale to the left refers to the long scan. For the short scan, both the 1st and the 2nd cycles are shown. Comparison between surfaces (b) coated with different nanoparticles sizes, (c) uncoated turned surface and coating with P25, and (d) different substrates. Sweep rate is at 50 mVs^−1^.

Cyclic voltammetry was used to characterise the electronic properties of the coated films. In Figure 3, examples of voltammograms are shown to illustrate the influence of scan range and number of scans for one type of electrodes (Figure 3(a)), different particle sizes (Figure 3(b)), partially coated electrodes (Figure 3(c)), and different substrates (Figure 3(d)).

The general features of the voltammograms obtained with Ti as substrate are the same with a symmetrical process at the most negative potentials and a peak at less negative potentials. The two different processes have been discussed in the literature in relation to films made of sintered TiO_2_ nanoparticles, where the electrolyte can penetrate the layer [1, 30–34]. The process observed at the most negative potentials has been attributed to charge accumulation in the conduction band (3) or filling traps just below the conduction band (4). In both cases, adsorption of protons takes place to obtain charge balance in acid solution [32]:

(3) TiIVO2+e−+H+⟷TiIVO2(eCB−)(H+)

(4) TiIVO2+e−+H+⟷TiIIIO2(H+)

In alkaline solutions, the electrolyte cation is probably the charge balancing species, that is, adsorption of K^+^ in the present case. The peak at less negative potentials has been attributed to filling of surface states below the conduction band, that is, reduction of Ti(IV) to Ti(III) according to (4). For reaction (4), the formation of TiO(OH) is also possible in alkaline solution. Alternatively, the peak at less negative potentials can be attributed to trap states at the grain boundaries in the film [1, 32]. Usually, the current at the most negative potentials increases exponentially, and eventually, hydrogen evolution takes place by reduction of water. The symmetry between cathodic and anodic charge observed for the nanoparticle-covered electrodes, TS + 8 nm and TS + 22 nm, indicates that faradaic processes are not involved if the potential is limited to −1.8 V. Polarising to more negative potentials results in further increase in the current due to hydrogen evolution (not shown). In contrast to other studies on nanoparticle-covered electrodes, the current goes through a maximum on the negative going scan, before hydrogen evolution sets in. The reason for this is not known but may be related to complete filling of energy levels just below the conduction band. The peak at less negative potentials has been attributed to filling of surface states or trap states at the grain boundaries in the film as mentioned above. In the present study, the films are not sintered, and the amount of grain boundaries is expected to be low and therefore the peak is most likely due to surface states. The positive potential limit was 0 V but this was not sufficient to completely emptying the surface bands filled during the negative going scan. This is evident from the much smaller peak observed on subsequent sweeps, Figure 3(a). The peak potential shifts depending on the coating with the most negative peak potential for the TS + 8 nm electrode followed by the TS + 22 nm and TS + P25 surfaces, Figure 3(b) and Table 2. Also the current for the process at more negative potentials depends on the type of particles, Figure 3(b), and has been related to the active surface area of nanoparticle films, indicating that the active surface area increases with decreasing particle size. For the native oxide always present on titanium metal, the reduction peak appears at less negative potentials (−0.89 V) compared with the particle-covered electrodes, and the redox process shows some reversibility with an oxidation peak at −0.81 V. Some reversibility in the redox process at these potentials is also observed for the TS + P25 electrode, Figure 3(c). The total charge for the naturally formed oxide is lower than for TS + P25, which is expected since the available surface area will be larger for the nanoparticle film of P25. A close look at the voltammogram for TS + P25 indicates that there is a contribution from the uncovered surface with naturally formed oxide (shown as a shoulder of the peak).

This is in agreement with the SEM image in Figure 2, where the uncovered surface is visible in the porous structure. The process at more negative potential is however the same and differs only in the reversibility with higher anodic charge for the TS + P25 electrode. The reversibility of this process is higher for the TS + 8 nm and TS + 22 nm electrodes compared to TS + P25 and the turned surfaces and is probably related to the smaller particle sizes and well defined surfaces. The lack of reversibility can be due to Faradaic processes and that is probably the case for the gold electrode covered with 22 nm TiO_2_ particles, Au + 22 nm. The reduction peak is closer to the conduction band, and hydrogen evolution is probably taking place at more negative potentials since no oxidation peak is observed, Figure 3(d). However, a small oxidation peak is observed at −0.82 V in close agreement with the peak observed for the Ti-coated electrodes. This peak is associated with emptying of energy states on the surface of the TiO_2_ particles.

Based on the voltammetric response and assuming that no Faradaic processes occur, the density of states (DOS) in the bandgap and the electron density can be determined using (5) [31, 33].

Let

(5) g0(−eE)=−(IeLAν)(eV),

where *g*_0_(−e*E*) is the first estimate of the DOS in the bandgap valid at zero Kelvin, *E* is the electrode potential, *L* the thickness of the layer, e the elemental charge, *A* the surface area, and *ν* the sweep rate. More elaborate equations are available to account for elevated temperature [35] but as a first approximation (6) will be used. The experimental data show two types of states, one associated with the exponential tail of the conduction band, *g*_tail_(−e*E*), at the most negative potentials, and states in the bandgap with a Gaussian-like distribution, *g*_gauss_(−e*E*), see (6) and (7).

Let

(6) gtail(−eE)=gtail,BEexp⁡[−αFERT],

where *g*_tail,*BE*_ is the DOS at the edge of the conduction band and *α* is related to extension of the tail into the bandgap [31, 33]. The Gaussian distribution is given in (7).

Let

(7) ggauss(−eE)=ggauss,sat·exp⁡[−(E−Ep)2/2σ2]σ2π,

where *g*_tail,*BE*_ corresponds to the complete filling of the states in the bandgap, *E*_*p*_ is the peak potential, and *σ* is the standard deviation about the peak potential. An attempt was made to fit experimental data to these equations but the states in the bandgap deviates from a Gaussian distribution, and reliable values could not be obtained. A more detailed analysis of the experimental data will be presented elsewhere.

For porous nanostructured films, the charge associated with the exponential increase has been shown to be proportional to the interfacial area [31]. Since the thicknesses of the coated layers are not known, the values extracted from the exponential term can only be used to calculate the interfacial area normalised with the area of the uncoated surface, Table 3. Comparing these values with the developed interfacial area, *S*_*dr*_, shows that even though P25 has the largest *S*_*dr*_ value the active area is only slightly higher than for the turned surface. In contrast, the smooth appearance of the TS + 8 nm and TS + 22 nm surfaces yields higher interfacial area with the largest area obtained for the smallest nanoparticles. The lack of agreement originates from the fact that *S*_*dr*_ represents the physical (passive) area while the interfacial (active) area is created in contact with the electrolyte.

#### 3.3.2. Electrochemical Impedance Spectroscopy

**Figure 4:**
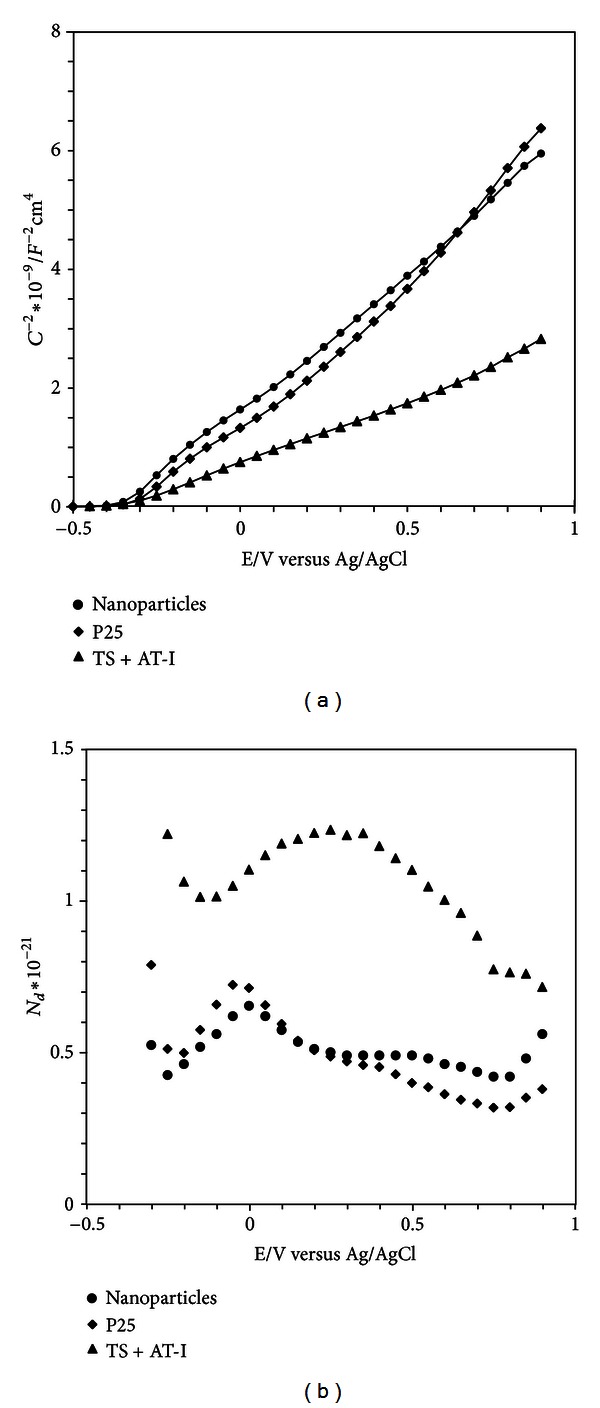
(a) The Mott-Schottky plots for different surfaces. The response for the TS + 8 nm and TS + 22 nm surfaces is the same and is shown as one graph. (b) The number of charge carriers.

In Figure 4, the Mott-Schottky plots for the different surfaces are shown. For Ti surfaces coated with 8 and 22 nm, the Mott-Schottky behaviour is the same, and only one curve is shown in the graph. For these surfaces, a linear Mott-Schottky relationship is found except close to the flat band potential, where a nonlinear behaviour is observed for all surfaces. For the TS + P25 and TS + AT-I surfaces, a change in the slope is observed at the most positive potentials. The number of charge carriers was calculated at each potential from the slope of the Mott-Schottky plot and is given in the inset to Figure 4. Despite changes in the slope of the Mott-Schottky curves, the number of charge carriers is fairly constant and the value for the most linear region is given in Table 3 together with the flat band potential. The flat band potentials were similar for the different surfaces, and the potentials are in good agreement with previously reported values [2, 36].

The potential drop over the oxide can be divided into two parts: one takes place over the less conducting outer part of the oxide and the other over the more conducting part closer to the metal/oxide interface [37–39]. Close to the flat band potential, most of the potential drop takes place over the insulating part, and it is possible to calculate the part of the potential, that is, used to establish the space charge region in the semiconducting part of the oxide [38]. It has been argued that for insulating films the sharp decrease of the *C* ^−2^ versus *E* curve close to the flat band potential is due to the capacitance of the oxide [38]. This is clearly the case for nanoparticle-covered gold electrodes and will be discussed further below.

For the surface covered with P25, the slope increases significantly at potentials higher than 0.4 V, giving a lower donor density by a factor of 1.6. This line can be extrapolated to yield an apparent flat band potential close to 0 V. Since the flat band potential is expected to be close to −0.35 V at the pH used in the present work, the apparent flat band potential is probably affected by surface states.

**Figure 5:**
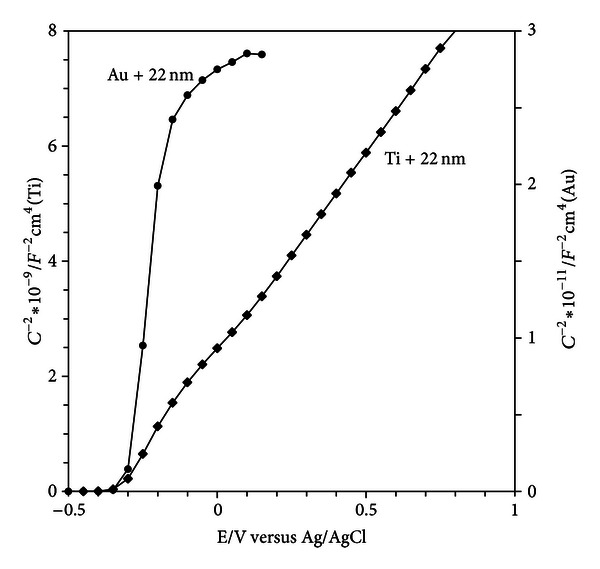
The Mott-Schottky plots obtained at 100 Hz. Note that the scale to the right is related to the Au + 22 nm electrode.

The Mott-Schottky plot for the Au + 22 nm electrode looks different compared with the other electrodes, Figure 5. In this case the capacitance was calculated from impedance values obtained at a constant frequency of 100 Hz.

For comparison, the Mott-Schottky curve for the TS + 22 nm electrode is also shown, with capacitance values calculated in the same way. Starting at positive potentials, the capacitance is constant for the Au + 22 nm surface, that is, independent of potential. This capacitance is therefore related to the capacitance of the insulating oxide film. Close to the flat band potential the curve changes rapidly. By correcting for the oxide capacitance, the donor density and the flat band potential can be estimated, Table 3. From the oxide capacitance value, the average oxide thickness can be calculated using (8). A value of 28 nm was obtained, which indicates that only a monolayer of particles is attached to the surface in case of gold.

Let

(8) d=ε0εrAC,

where *ε*_0_ is the dielectric permittivity of vacuum, *ε*_*r*_ is the dielectric constant of TiO_2_  (*ε*_*r*_ = 60),  *A* is the electrode area, and *C* is the capacitance.

The number of charge carriers is much lower for the nanoparticles film on gold than for the same film on titanium. This indicates that the TiO_2_ nanoparticles are interacting more closely with the thin oxide film on titanium than with the gold metal. As a consequence, it is tempting to conclude that the surface states observed in cyclic voltammetry originate not only from the particles but also from the interface between the particles and the native oxide film on Ti. For the TS + AT-I surface, the number of charge carriers is higher than for the nanoparticle films and also higher than for the native oxide (TS), Table 3. One reason for the slightly higher conductivity could be the presence of titanium hydride in the metal phase. However, for blasted samples, the conductivity for surface with the AT-I treatment was found to be lower than for the blasted sample [16].

**Figure 6:**
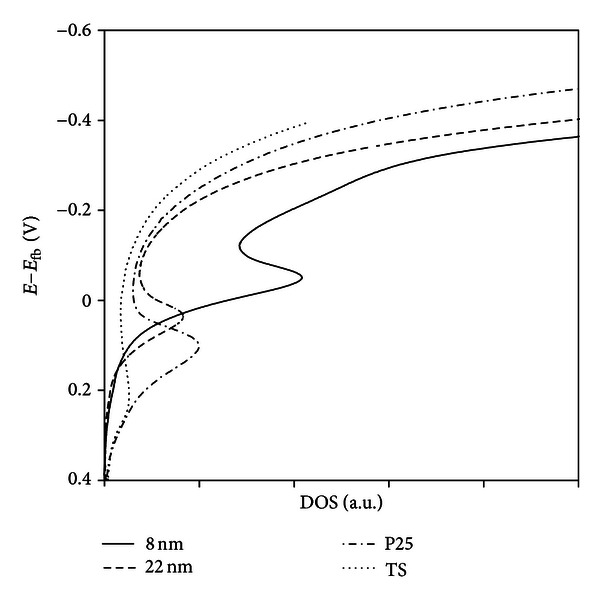
Density of states calculated using (6) with the layer thickness constant equal to 3 nm, which is the thickness of the naturally formed film. The relative DOS values are affected by the thickness of the layer.

The voltammetric measurements were made in alkaline solution and by using the experimentally determined flat band potentials obtained in acid solution and assuming a Nernstian pH shift, the flat band potential was calculated for pH 13, *E*_fb_ = −1.1 ± 0.1 V. In Figure 6, the density of states is plotted as a function of potential with reference to the flat band potential.

For the surface with 8 nm particles, the maximum in DOS occurs at higher energies than the flat band potential, while for the other surfaces the maximum is close to *E*_fb_ (TS + 22 nm) and at positive potentials with respect to *E*_fb_ (TS + P25 and TS). The location of the energy bands may be important for adsorption of bioactive compounds and in turn for the surface ability to function *in-vivo*. This will be further discussed below in relation to the result obtained after immersion in simulated body fluid.

### 3.4. Immersion in Simulated Body Fluid (SBF)

The ability of the surfaces to induce apatite nucleation was evaluated by immersion samples in SBF solution for 12 h, 72 h, and 1 week. The SBF solution contains ions with similar concentration as the human blood plasma and the recipe for SBF solutions can vary. In the present paper, the revised SBF recipe presented by Oyane et al. [26] was used. The samples were mounted with the treated surface hanging upside down to prevent gravitational precipitation.

The amount of apatite formed was measured by EDX. The apatite coverage (Θ) was calculated from the ratio of the titanium signal after and before immersion in SBF solution using (9). It is assumed that apatite is the only precipitate formed during immersion.

Consider

(9) Θ=1−(TiSBFTi).

**Figure 7:**
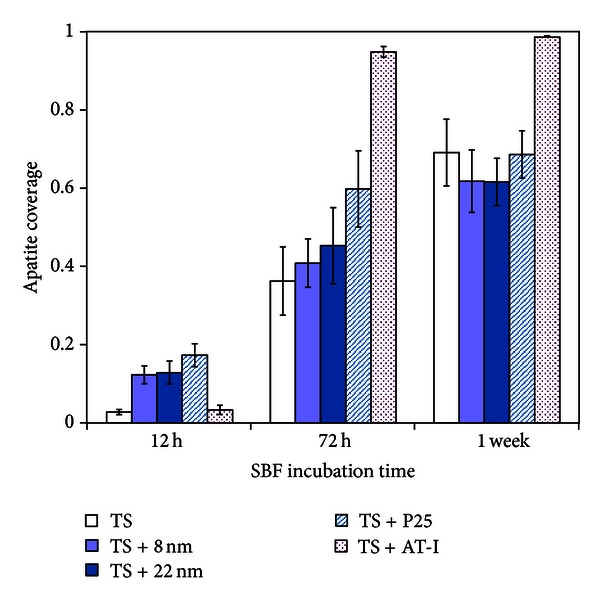
Apatite coverage on different surfaces after immersion in SBF solution for 12 h, 72 h, and 1 week.

When analyzing data for all surface groups immersed in SBF for 12 h with one-way ANOVA analysis (SPSS Inc, Chicago, USA), there was a statistically significant difference (*P* < 0.001) between surface groups. The results are shown in Figure 7, and it is interesting to note that the early nucleation of apatite is higher for surfaces coated with TiO_2_ nanoparticles compared to the reference (TS) and the TS + AT-I surface. Early nucleation of apatite crystals has been reported for surfaces with anatase structure as compared to an amorphous or rutile structure [40–42]. This could be one explanation for the early nucleation rate observed for the coated surfaces, since the surface particles are predominantly of anatase phase [18, 43]. The experimental data also show that the active surface area increases with decreasing particle size, Table 3. A higher surface area provides more nucleation sites and may facilitate the deposition of apatite. Also the form of the nanosized deposits seems to have an influence on the early nucleation since the AT-I surface, with rod like deposits, has similar active area as the TS + 22 nm surface but different nucleation rate. It is also worth mentioning that the electric properties, that is, number of charge carriers, seem to correlate with the early nucleation. Both TS and TS + AT-I have higher *N*_*d*_ that the nanoparticle-covered surfaces. After 72 h immersion in SBF, the difference between the reference and the coated surfaces (TS + 8 nm and TS + 22 nm) has disappeared (*P* = 0.30 when compared with one-way ANOVA). Instead, the TS + AT-I surface shows the highest apatite coverage (*P* < 0.001 when compared to TS + P25 in a Student's *t*-test). This trend is maintained after 1 week (Figure 7), when the TS + AT-I surface shows the highest coverage degree (*P* < 0.001) compared to the reference and coated surfaces, among which no differences are found (*P* = 0.082 when the groups are compared with a one-way ANOVA analysis).

**Figure 8:**
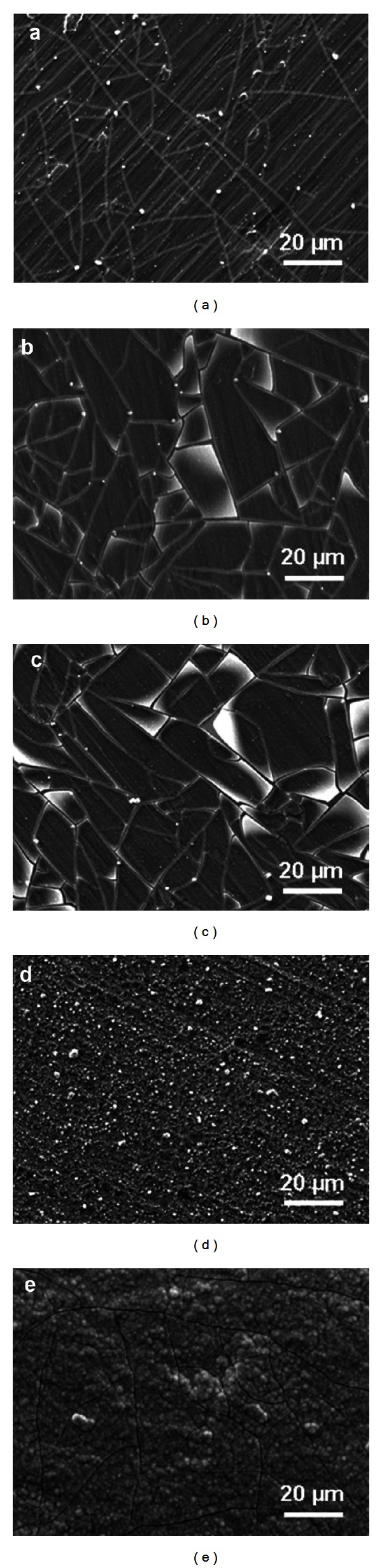
SEM images after immersion in SBF solution for 1 week. Titanium discs with (a) turned surface (TS) and (b)–(d) coated surfaces with 8 nm (TS + 8 nm), 22 nm (TS + 22 nm), and P25 (TS + P25) particles. (e) Shows turned surface treated with oxalic acid and diluted hydrofluoric acid (TS + AT-I).

The morphology of the different surfaces was investigated by SEM, and clear differences were obtained, Figure 8. After 1 week immersion in SBF, thin apatite layers were formed on the reference and coated surfaces leaving the underlying surface still visible. For the TS + AT-I surface, the underlying surface was completely covered by a thick apatite layer, and this is also confirmed by the EDX measurements, Figure 7. The influence of surface roughness on apatite formation has been previously investigated and with surface roughness corresponding to an *R*_*a*_ value between 0.2 and 0.6 *μ*m a continuous and adherent apatite layer has been shown to form on various materials [40, 44, 45]. It is difficult to compare absolute values of roughness parameters due to differences in measuring techniques and analysis. However, relative values can be used, and the results after 1 week of immersion support the previous findings that rougher surfaces favour formation of thick and adherent apatite layers compared to smoother surfaces, Table 3 and Figure 8.

**Figure 9:**
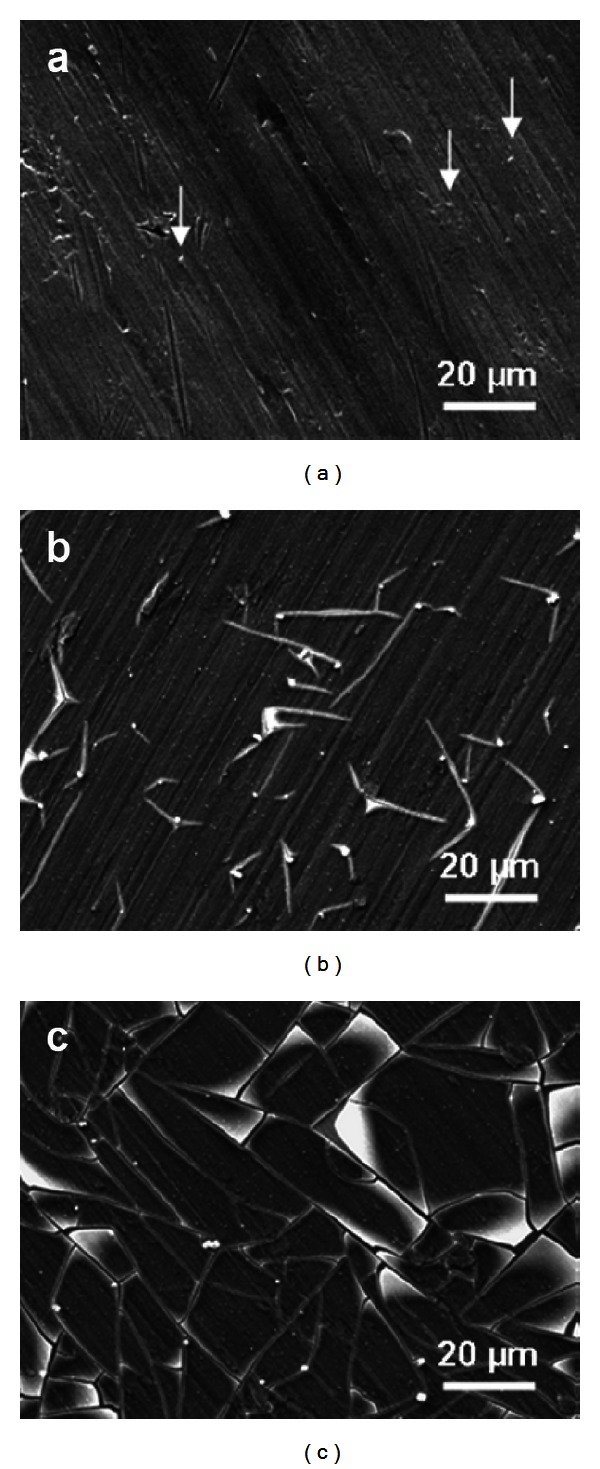
SEM images of the TS + 22 nm surface after immersion in SBF solution for (a) 12 h, (b) 72 h, and (c) 1 week. Distinct precipitates were observed after 12 h (marked with arrows).

Fractured apatite layers with different characteristics were observed for all surfaces except for the TS + P25 surface. Fractures formed on TS + AT-I (Figure 8(e)) after one week of immersion resembled fractured apatite layers formed on TiO_2_ coatings [46], *α*-CaSiO_3_ ceramics [47], chemically treated titanium [48], and alkali- and heat-treated titanium [49]. The apatite layer formed on the TS + 8 nm and TS + 22 nm surfaces (Figures 8(b) and 8(c)) exhibited another type of fractures similar to those on apatite layers formed on titanium, first incubated in a fibronectin solution and thereafter in Hank's Buffer Saline Solution (HBSS) for one week [50]. The cracks or fractures have been suggested to be caused by drying shrinkage [40, 51] and have been observed to be larger and deeper with increasing immersion time [40, 51]. An alternative explanation is related to the 3D growth mechanism for thick apatite layers, where nucleus on the surface grows and eventually forms a fully covering film. When the different nuclei start to interact, stresses are imposed and the apatite layer cracks [41, 52]. This mechanism seems to be valid for the TS + AT-I surface with no precipitates after 12 h but a fully covering film after 72 h. The mechanism of nucleation seems to be different for the surfaces with nanoparticles. The fast precipitation of hydroxyapatite at certain sites on the surface is followed by 2D growth, where the layer has weak interaction with the underlying substrate. The formation of a 2D layer seems to prevent the formation of bigger agglomerates commonly observed and as a consequence the layer remains thin. The early nucleation observed on surfaces covered with nanoparticles is illustrated for the TS + 22 nm surface in Figure 9. After 12 h, distinct precipitates are observed (marked with arrows in Figure 9(a)) but the surface is not fully covered by precipitates. The Ca/P ratio of the precipitates at this stage is close to 1.7, and no Ca and P signals were obtained in between the precipitates. This indicates, formation of hydroxyapatite (Ca_5_(PO_4_)_3_(OH)), which is the thermodynamically most stable phase [53]. However, the rate of formation for hydroxyapatite has been reported to be much slower than for other apatite compounds [54]. Hydroxyapatite is the main mineral in bone and is crucial to obtain high mechanical strength [55]. After 72 h of immersion, the surface is covered with a thin apatite layer and cracks start to form, Figure 9(b). It seems like the crack propagates from the initial precipitates due to stresses induced by the miss match between the precipitates and the layer. After one week of immersion, the apatite layer is fully developed but still thin, Figure 9(c). In the present study, all surfaces (except TS + P25) had fractures irrespective of the surface roughness, Table 3 and Figure 8. However, the apatite layers of the TS + 8 nm and TS + 22 nm surfaces seem to be detaching from the underlying surface. EDX measurements performed within a crack in the apatite layer show no or only traces of Ca and P. Detachment of apatite layers has been reported elsewhere [44, 56] and are believed to be caused by weak interactions with the underlying substrate and the absence of surface roughness. Also in the study by Leitão et al. [45], detachment of apatite layers was more pronounced on smooth surfaces than on rough surfaces.

The Ca/P ratio of the formed apatite films was calculated from the EDX measurements and was found to range between 1.42 and 1.56 for all surfaces after 72 h and 1 week immersions. This could indicate the formation of tricalcium phosphate (Ca_3_(PO_4_)_2_) with Ca/P ratio = 1.5. However, a Ca/P ratio of ~1.5 has also been reported for amorphous layers [57], and it has been shown that the Ca/P ratio depends on the synthesis conditions with values ranging from 1.3 to 1.8 [53].

**Figure 10:**
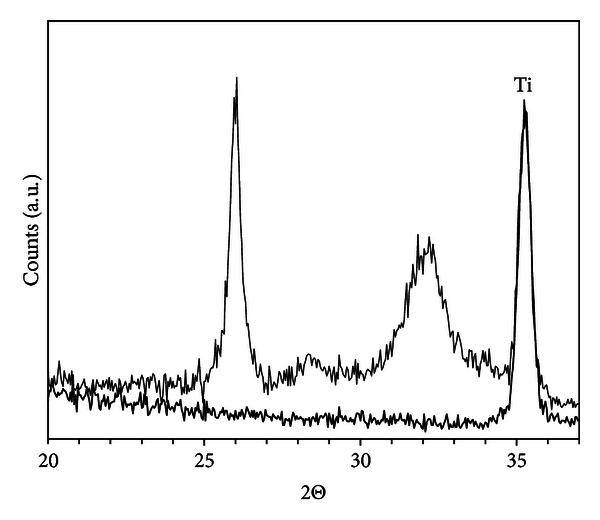
X-ray diffraction on TS + AT-I before (thick line) and after (thin line) immersion in SBF solution. The diffraction peaks at 2Θ values 26, 28, and 32° correspond to crystalline hydroxyapatite. The diffraction spectra are normalised to the titanium peak at 2Θ = 35.

The chemical composition of the formed apatite layers was analysed by gracing angle-X-ray diffraction (GI-XRD). Although the EDX measurements show rather high apatite coverage for the TS, TS + 8 nm, TS + 22 nm, and TS + P25 surfaces after 1 week, only weak and broad diffraction signals were obtained. This indicates that the layers formed are amorphous, which is also supported by the Ca/P ratio 1.5. Homogeneous growth from an SBF solution has been shown to start with the formation of an amorphous phase with subsequent formation of small apatite crystals [53]. For the TS + AT-I surface, where thicker apatite layers were obtained, clear diffraction peaks for hydroxyapatite [58] are observed after 72 h and 1 week of immersion in SBF, Figure 10.

The long term results show that on a rougher surface the development of thick apatite layers is facilitated. However, the smoother surfaces containing nanoparticles show faster nucleation and formation of thin 2D layers of amorphous apatite. The correlation between a surface ability to nucleate hydroxyapatite and *in-vivo* response has been recently reviewed [59] and challenged [60]. Surfaces with poor ability to nucleate apatite are shown to work well *in-vivo* and vice versa [60]. It can be argued that some of the inconsistency originates from different apatite formation mechanisms.

## 4. Conclusions

The electronic properties of TiO_2_ nanoparticle films on titanium and gold differ significantly with lower conductivity for the film on gold. The reason for this is probably a stronger interaction between the native oxide on titanium and the nanoparticles as compared with the gold nanoparticle interaction. Differences in the electronic structure of the different nanoparticle films are also visible in cyclic voltammetry. The peak related to surface states with energy bands in the bandgap of TiO_2_ moves closer to the conduction band as the donor density in the film decreases. The donor density is calculated from the impedance measurements and related to the active surface area determined from cyclic voltammetry. The trend in the active surface area is different from the trend observed for the developed interfacial area, *S*_*dr*_, which illustrates the differences between the physical area and the area related to the semiconducting properties of the oxide.

Immersion in SBF solution can be a measure of the bioactivity of different surfaces. In the present study, two types of nucleation and growth behaviours were observed. For the rougher surfaces, the nucleation is initially delayed but once it starts, thick layers are formed. These layers have cracks induced by stresses in the film formed when different growing nuclei coalesce. For the smoother surfaces with small anatase nanoparticles, the initial precipitation is fast but only few small nuclei of hydroxyapatite are formed leaving the rest of the surface uncovered. On these surfaces, an amorphous 2D film develops with a Ca/P ratio close to 1.5. The growth of this layer is slow and after one week of immersion the underlying surface structure is still visible through the apatite layer. The influence of different nucleation mechanisms on the biocompatibility is not known but would be of great interest to explore.

For surface films formed from small well dispersed nanoparticles, a porous layer is created on the surface. This results in both a larger donor density as determined from the impedance measurements and a larger active area compared with the turned surface and the surface containing agglomerates of P25. For surfaces with similar roughness, the ability to nucleate apatite is lower for less conducting oxide layers as shown here where for the more conducting surface film TS + AT-I a thick apatite layer is formed. This surface film also has a large developed surface area. The physical surface roughness seems to have an important role in the apatite formation since for the reference surface (TS), with rather high conductivity, apatite formation is limited. For the early nucleation, the nanoparticle-covered surfaces seems to be preferred.

The high number of energy states in the bandgap found for the nanoparticles surfaces may be beneficial for adsorption of redox active proteins such as fibrinogen and will be investigated in a forthcoming study.

## Figures and Tables

**Table 1:** Sample description.

Sample name	Underlying surface	Surface modification
TS	Turned titanium	None
TS + 8 nm	Turned titanium	Spin coated with 8 nm TiO_2_ particles
TS + 22 nm	Turned titanium	Spin coated with 22 nm TiO_2_ particles
TS + P25	Turned titanium	Spin coated with P25 particles
TS + AT-I	Turned titanium	Treated sequentially in oxalic and hydrofluoric acid [16]
Au	Mechanically polished gold	None
Au + 22 nm	Mechanically polished gold	Spin coated with 22 nm TiO_2_ particles

**Table 2:** 3D surface roughness parameters determined by AFM.

Sample	*S*_*a*_ (nm)		*S*_*dq*_		*S*_*dr*_ (%)	
	Average	Stdev	Average	Stdev	Average	Stdev
TS	11.6	0.67	0.26	0.09	3.51	2.06
TS + 8 nm	8.34	—	0.14	—	1.05	—
TS + 22 nm	10.6	1.4	0.16	0.01	1.32	0.20
TS + P25	63.3	9.2	1.73	0.26	109.3	24.5
TS + AT-I	41.8	6.2	0.89	0.23	38.3	19.2
Au	13.9	—	0.24	—	2.68	—
Au + 22 nm	9.02	1.8	0.14	0.02	1.00	0.70

*S*_*a*_: average height of surface, *S*_*dr*_: developed interfacial area, *S*_*dq*_: root mean square of surface slope. Data from 10 × 10 *μ*m scan size a Gaussian filter of 20% of the horizontal width (1.996 *μ*m).

**Table 3:** Peak potentials determined from CV, *E*_fb_, and *N*_*d*_ determined from EIS.

Sample	^ a^ *E*_*p*_/V	^ b^Rel. active area (A_aa_)	^ c^ *E*_fb_/V	^ c^ *N*_*d*_×10^−19^/cm^−3^	^ d^ *N*_*d*_×10^−19^/A_aa_
TS	−0.89	1	−0.35^d^	84^d^	84
TS + 8 nm	−1.16	2.65	−0.30	47	17.7
TS + 22 nm	−1.09	1.77	−0.30	47	26.6
TS + P25	−1.00	1.30	−0.35	50	38.5
TS + AT-I	−0.93	1.88	−0.41	119	63.3
Au + 22 nm	−1.27	—	−0.33	0.13	—

^a^ *E*_*p*_ is determined in 0.1 M KOH. ^b^Calculated on the exponential term for *E* = 0 – (−1.5) V. ^c^ *E*_fb_ and *N*_*d*_ are determined in 0.5 M H_2_SO_4_. ^d^From [17], recalculating the value for *E*_fb_ to the pH is used in this study.
